# Integrated analysis of dysregulated long non-coding RNAs/microRNAs/mRNAs in metastasis of lung adenocarcinoma

**DOI:** 10.1186/s12967-018-1732-z

**Published:** 2018-12-27

**Authors:** Lifeng Li, Mengle Peng, Wenhua Xue, Zhirui Fan, Tian Wang, Jingyao Lian, Yunkai Zhai, Wenping Lian, Dongchun Qin, Jie Zhao

**Affiliations:** 1 Department of Pharmacy, The First Affiliated Hospital of Zhengzhou University, Zhengzhou, 450052 Henan China; 2 Department of Clinical Laboratory, The Third People’s Hospital of Henan Province, Zhengzhou, 450052 Henan China; 3 Cancer Center, The First Affiliated Hospital of Zhengzhou University, Zhengzhou, 450052 Henan China; 4 Department of Clinical Laboratory, The First Affiliated Hospital of Zhengzhou University, Key Laboratory of Laboratory Medicine of Henan Province, Zhengzhou, 450052 Henan China; 5 National Engineering Laboratory for Internet Medical Systems and Applications, Zhengzhou, 450052 Henan China

**Keywords:** Lung adenocarcinoma, Metastasis, Biomarker, lncRNAs

## Abstract

**Background:**

Lung adenocarcinoma (LUAD), largely remains a primary cause of cancer-related death worldwide. The molecular mechanisms in LUAD metastasis have not been completely uncovered.

**Methods:**

In this study, we identified differentially expressed genes (DEGs), miRNAs (DEMs) and lncRNAs (DELs) underlying metastasis of LUAD from The Cancer Genome Atlas database. Intersection mRNAs were used to perform gene ontology (GO), Kyoto Encyclopedia of Genes and Genomes (KEGG) pathway and co-expression network analysis. In addition, survival analyses of intersection mRNAs were conducted. Finally, intersection mRNAs, miRNAs and lncRNAs were subjected to construct miRNA-mRNA-lncRNA network.

**Results:**

A total of 1015 DEGs, 54 DEMs and 22 DELs were identified in LUAD metastasis and non-metastasis samples. GO and KEGG pathway analysis had proven that the functions of intersection mRNAs were closely related with many important processes in cancer pathogenesis. Among the co-expression interactions network, 22 genes in the co-expression network were over the degree 20. These genes imply that they have connections with many other gene nodes. In addition, 14 target genes (ARHGAP11A, ASPM, HELLS, PRC1, TMPO, ARHGAP30, CD52, IL16, IRF8, P2RY13, PRKCB, PTPRC, SASH3 and TRAF3IP3) were found to be associated with survival in patients with LUAD significantly (log-rank P < 0.05). Two lncRNAs (LOC96610 and ADAM6) acting as ceRNAs were identified based on the miRNA-mRNA-lncRNA network.

**Conclusions:**

Taken together, the results may provide a novel perspective to develop a multiple gene diagnostic tool for LUAD prognosis, which might also provide potential biomarkers or therapeutic targets for LUAD.

**Electronic supplementary material:**

The online version of this article (10.1186/s12967-018-1732-z) contains supplementary material, which is available to authorized users.

## Background

Lung cancer is the leading cause of cancer-related deaths worldwide, despite advances in lung cancer therapy, the average 5-year survival rate is only 18% [1, 2]. A majority of the deaths associated with lung cancer are due to secondary disease or metastatic progression [3]. Lung adenocarcinoma (LUAD) is a major lung cancer that is in a locally advanced or metastatic stage at the time of diagnosis, which leaves no time for early detection or treatment [4]. An early and accurate diagnosis may warrant timely treatment to potentially decrease the mortality. Therefore, molecular mechanisms that may help understand metastases of LUAD should be investigated to contribute to early diagnosis, better treatment and better overall prognosis of this disease.

Numerous published articles demonstrate that dysregulated genes are essential for initiation and progression of lung cancer. In recent years, the development of microarray technology has served as an effective measure to identify differentially expressed genes [5], and provides new insight into the alteration of gene expression during tumorigenesis [6]. Differentially expressed genes can be found through different experimental treatments, and their biological functions can be speculated via known information. The application of high-throughput miRNA profling methods, such as RNA sequencing and microarrays, has enabled researchers to identify a group of miRNAs as biomarkers in cancer diagnosis [7]. MicroRNAs (miRNAs) are a class of single stranded, non-coding RNAs of 19–25 nucleotides [8], which transcriptionally or post-transcriptionally regulate gene expression through binding to targeted mRNAs and influence the degradation and translation of mRNA [9]. Accumulating evidence suggests that aberrant levels of miRNAs are linked to proliferation, angiogenesis, and metastasis in various human malignancies [10]. Besides, miRNA has incurred broad attention as a targeting choice in cancer therapies [11] or as the diagnostic or prognostic markers [12]. Long non-coding RNAs (lncRNAs) are non-coding RNAs ranging in length from 200 nucleotides to ~ 100 kb [13]. LncRNAs have mechanistically diverse functions in the cell, and in the nucleus, LncRNA has been shown to regulate gene expression in either cis or trans by recruiting a chromatin-modifying complex to the promoter of a target gene [14, 15]. Besides, it has been reported that lncRNAs are key competing endogenous RNAs (ceRNAs) harboring miRNA response elements (MREs) and serve as ceRNAs to exchange with mRNAs via competitively binding to common miRNAs [16].

The Cancer Genome Atlas (TCGA) is one prominent example of the renowned public databases which provides a platform of RNA sequencing with mRNA, miRNA and lncRNA data of various cancers. By integratively analysis RNA-Seq and miRNA-Seq data of LUAD samples from TCGA database, we successfully corhorted a set of differentially expression genes (DEGs), miRNAs (DEMs) and lncRNAs (DELs) underlying LUAD metastasis and non-metastasis samples. Based on intersection mRNAs, gene ontology (GO), Kyoto Encyclopedia of Genes and Genomes (KEGG) pathway enrichment and co-expression analysis were conducted. Furthermore, we performed a receiver operating characteristic (ROC) analysis to investigate the diagnostic value of intersection mRNAs. Finally, miRNA-mRNA-lncRNA network were constructed. Our study might provide a meaningful contribution to exploring the mechanisms of LUAD metastasis and candidate diagnostic biomarkers and therapeutic targets.

## Materials and methods

### DEGs, DEMs and DELs of LUAD metastasis and non-metastasis samples from TCGA data

The LUAD metastasis and non-metastasis RNA-Seq and miRNA-Seq data were downloaded from the TCGA database using The GDC Data Portal (https://gdc-portal.nci.nih.gov/). The mRNA and lncRNA expression data included a total of 372 samples consisting of 25 LUAD metastasis and 347 non-metastasis samples. The miRNA expression data included a total of 305 samples consisting of 19 LUAD metastasis and 286 non-metastasis samples. The clinical characteristics of the 384 TCGA patients are shown in Table 1 (Additional file 1). No ethical issues were involved, because the sequencing data were obtained by using TCGA database. The edgeR package in Bioconductor was used to screen the DEGs, DEMs and DELs in LUAD metastasis and non-metastasis samples. The genes, miRNAs and lncRNAs were considered as DEGs, DEMs and DELs if P-value < 0.05 (including up-regulation and down-regulation), respectively (Additional file 2).

**Table 1:** Clinical characteristics of TCGA cohort

	All patients (n = 372)
Age at diagnosis (years)	
Mean	65
Range	38–85
Sex	
Male	183 (49.2%)
Female	189 (50.8%)
Clinical stage	
I	3 (0.8%)
Ia	85 (22.9%)
Ib	101 (27.2%)
IIa	34 (9.1%)
IIb	53 (14.2%)
IIIa	55 (14.8%)
IIIb	10 (2.7%)
IV	25 (6.7%)
NA	6 (1.6%)
Metastasis	
Yes	25 (6.7%)
No	347 (93.3%)
Overall survival	
Deaths	148 (39.8%)
Alive	224 (60.2%)
Follow-up (months)	
Mean	30.8
Range	0–238.3

### Intersection lncRNAs and mRNAs

Predicted DEMs targets in this study were determined using miRBase targets (http://mirdb.org/miRDB/) to predict target genes, and using miRanda (http://www.microrna.org/microrna/home.do) to find the lncRNA-miRNA interactions. Then we combined the information of miRNAs predicted and differentially expressed data of TCGA by using Excel to obtain the repeating part to choose the intersection lncRNAs and mRNAs.

### GO and KEGG pathway enrichment analysis

Differentially expressed intersection mRNAs were obtained from the Database for Annotation, Visualization and Integrated Discovery (DAVID), which provided a comprehensive set of functional annotation tools for investigators to understand the biological meaning behind large lists of genes (http://david.abcc.ncifcrf.gov/). Up and down-regulated genes were analyzed, respectively. Two-side Fisher’s exact test and *χ*^2^ test were used to classify the GO category, and the false discovery rate (FDR) was calculated to correct the P-value,the smaller the FDR, the smaller the error in judging the p-value [17]. The FDR was defined as $FDR = 1 - \frac{{N_{k} }}{T}$, where *N*_*k*_ refers to the number of Fisher’s test P-values less than *χ*^2^ test P-values. We computed P-values for the GOs of all the differential genes. Enrichment provides a measure of the significance of the function: as the enrichment increases, the corresponding function can be more specific, which helps us to find those GOs with more concrete function description in the experiment. Within the significant category, the enrichment Re was given by: Re = (*n*_*f*_/*n*)/(*N*_*f*_/*N*) where “*n*_*f*_” is the number of flagged genes within the particular category, “*n*” is the total number of genes within the same category, “*N*_*f*_” is the number of flagged genes in the entire microarray, and “*N*” is the total number of genes in the microarray [18].

KEGG (http://www.kegg.jp/) was used to analyze the potential functions of these genes participated in the pathways [19]. Still, we turn to the Fisher’s exact test and *χ*^2^ test to select the significant pathway, and the threshold of significance was defined by P-value and FDR. The enrichment Re was calculated like the equation above [20–22]. P < 0.05 was considered as the threshold criterion.

### Co-expression analysis

Differentially expressed intersection mRNAs which had similar expression profiles were included in the differentially co-expressed genes analysis to investigate the potential metastatic mechanism of LUAD. The differentially co-expressed genes in LUAD metastasis samples compared to non-metastasis samples were identified via the DCGL package in R. In the network analysis, a degree is the most important parameter of the centrality of a gene within a network that determines the relative importance. Up-regulated and down-regulated mRNAs were showed in pies with different colors. Then, the differential co-expression network was constructed based on the Cytoscape software.

### Survival analysis

The univariable Cox regression analysis was performed to assess the relationship between mRNAs expression levels and LUAD patients overall survival (OS) and recurrence free survival (RFS) time and identify survival-associated genes. The survival curves were plotted and tested using the Kaplan–Meier method and log-rank test. According to the median risk score, LUAD patients were divided into high- and low-risk groups. The mRNAs with log-rank P < 0.05 between high-risk and low-risk groups were considered statistically significant. R software and Bio-conductor were used for all these analyses. Next, we get target genes by combining significant survival-associated genes and genes incorporated into the gene co-expression network.

### Receiver operating characteristic (ROC) curve analysis

In order to assess the diagnostic value of target genes in LUAD, receiver operating characteristic (ROC) analysis were performed using pROC package in R language [23]. Diagnostic ability of the prediction model was evaluated, by calculating the area under a ROC curve. The ROC curve was used for classifier evaluation, and was drawn by plotting sensitivity against the false-positive rate. The area under the curve (AUC) under binomial exact confidence interval was calculated to generate the ROC curve.

### MiRNA-mRNA-lncRNA network

According to the relationship among mRNA, miRNA and lncRNA, the posttranscriptional regulation of mRNA transcripts bound by single-stranded miRNAs is basically established. We have established a theory based on lncRNA regulating miRNA abundance by isolating and binding them, acting as a so-called miRNA sponge [24]. Then, according to the theory of ceRNA, we chose the miRNA negatively regulated expression of lncRNAs and mRNAs to construct the miRNA-mRNA-lncRNA network. The miRNA-mRNA-lncRNA network was constructed and visualized using Cytoscape v3.0 [25].

### Statistical analysis

All data in the present study were analyzed using GraphPad Prism 6.0 software. Mean ± standard deviation and independent-samples t-test was used in the statistical analysis. A P-value less than 0.05 was considered statistically significant.

## Results

### Workflow

**Fig. 1:**
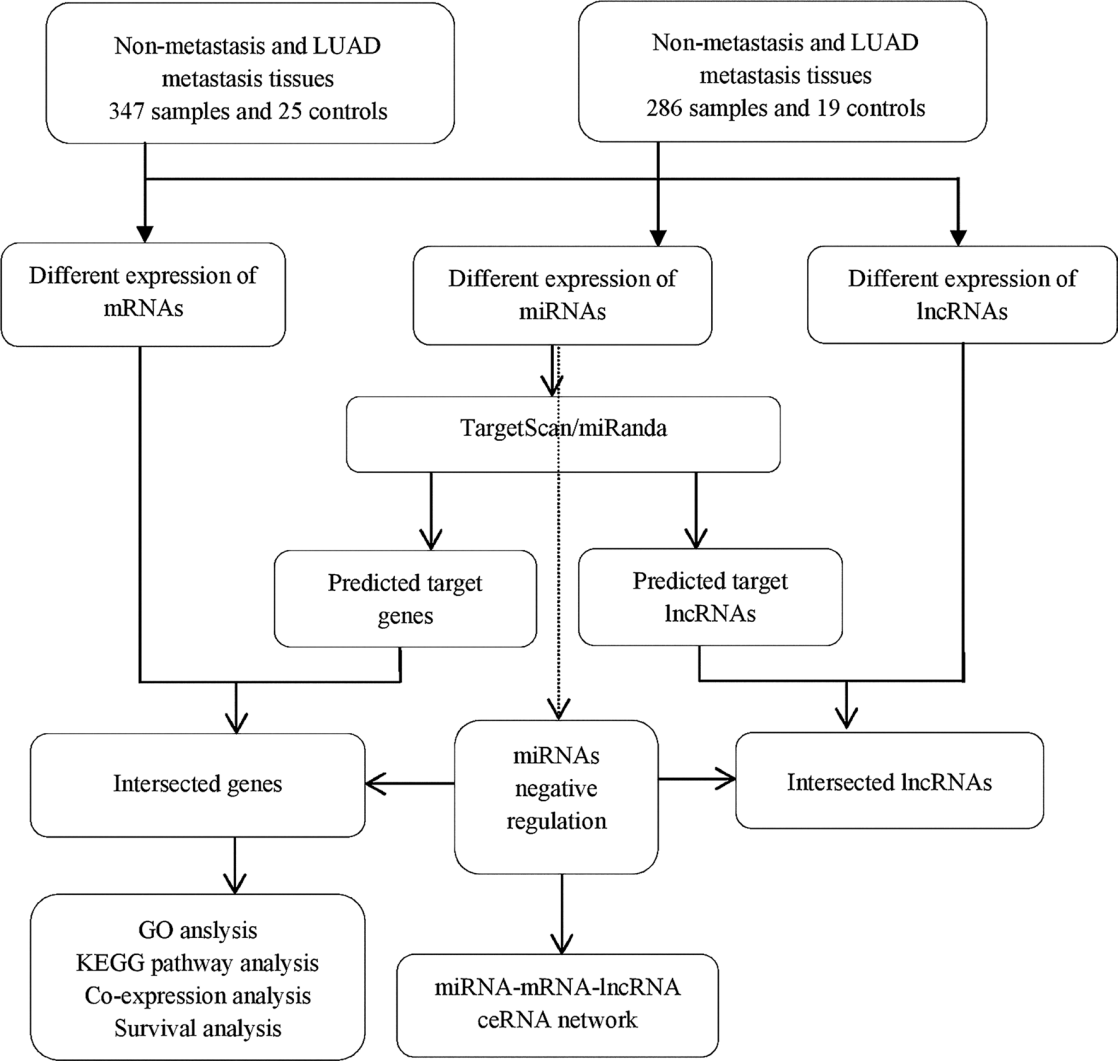
Flow chart of bioinformatics analysis

The present study consists of several processes sequentially (Fig. 1), that is TCGA-based RNA-seq data aggregation, and multiple bioinformatics analyses.

### DEGs, DEMs and DELs in LUAD metastasis and non-metastasis samples based on TCGA data

**Fig. 2:**
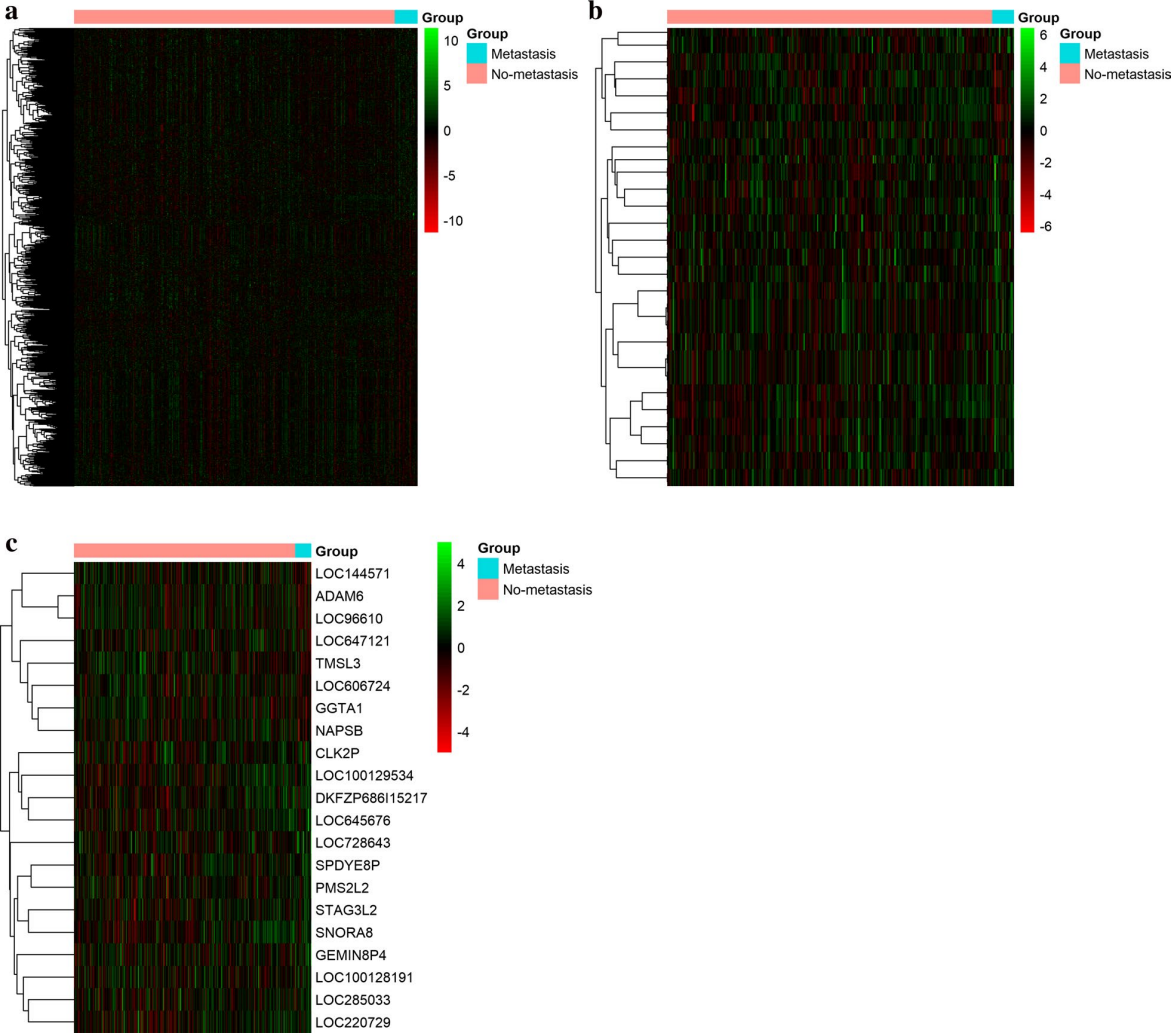
Heatmap for hierarchical cluster analysis of DEGs, DEMs and DELs expression levels change between LUAD metastasis and non-metastasis tissues (**a**–**c**). Colors ranged from green (low expression) to red (high expression), representing the relative expression levels of DEGs, DEMs and DELs

A total of 1019 genes were considered as DEGs in LUAD metastasis samples compared to non-metastasis samples, including 581 (57.0%) up-regulated and 438 (43.0%) down-regulated genes. And the top 20 DEGs in LUAD metastasis samples compared with non-metastasis samples were shown in Table 2. 54 miRNAs were considered as DEMs, including 12 (22.2%) up-regulated and 42 (77.8%) down-regulated miRNAs. 21 lncRNA s were considered as DELs, including 8 (38.1%) up-regulated and 13 (61.9%) down-regulated lncRNAs (Fig. 2).

**Table 2:** The top 20 DEGs in LUAD metastasis samples compared with non-metastasis samples

Gene name	P-value	Geom mean of intensities in LUAD non-metastasis	Geom mean of intensities in LUAD metastasis	log2 (FC)	Style
ADRA2A	2.77E−03	138.17	57.40	1.27	Up
SFTPB	3.64E−02	57,876.78	24,815.46	1.22	Up
IGJ	1.94E−03	6335.86	2744.20	1.21	Up
CD79A	2.31E−03	365.56	160.15	1.19	Up
CPZ	2.26E−05	264.00	119.83	1.14	Up
CH25H	4.05E−03	140.60	68.34	1.04	Up
LRRC15	1.60E−02	271.70	133.16	1.03	Up
UCHL1	4.55E−02	380.51	784.12	− 1.03	Down
SFRP2	1.63E−02	1526.84	757.42	1.01	Up
SKAP1	1.25E−03	138.21	69.33	0.99	Up
RNASE1	2.25E−03	7830.26	3995.57	0.97	Up
CILP2	9.77E−03	146.06	74.91	0.96	Up
LTB	1.57E−03	264.47	139.19	0.93	Up
COL10A1	1.65E−02	682.85	363.03	0.91	Up
TRIM16L	9.68E−03	184.04	342.52	− 0.89	Down
DERL3	1.83E−03	602.72	327.93	0.88	Up
CPXM1	2.46E−03	209.17	113.48	0.88	Up
APBA2	1.29E−02	220.67	121.83	0.86	Up
PODN	7.01E−04	746.37	414.51	0.85	Up
FMO3	1.62E−03	165.01	92.10	0.84	Up

### Intersection lncRNAs and mRNAs

The mRBase targets method was used to analyse the target mRNAs of DEMs and obtained the 915 miRNA targeted mRNAs. The miRanda method was used to analyse the target lncRNAs of DEM and obtained the 22 miRNA-targeted lncRNAs. Then the study combined the information of miRNAs predicted and differentially expressed data of TCGA, and obtain 915 intersection mRNAs and 20 intersection lncRNAs.

### GO and KEGG analysis

**Fig. 3:**
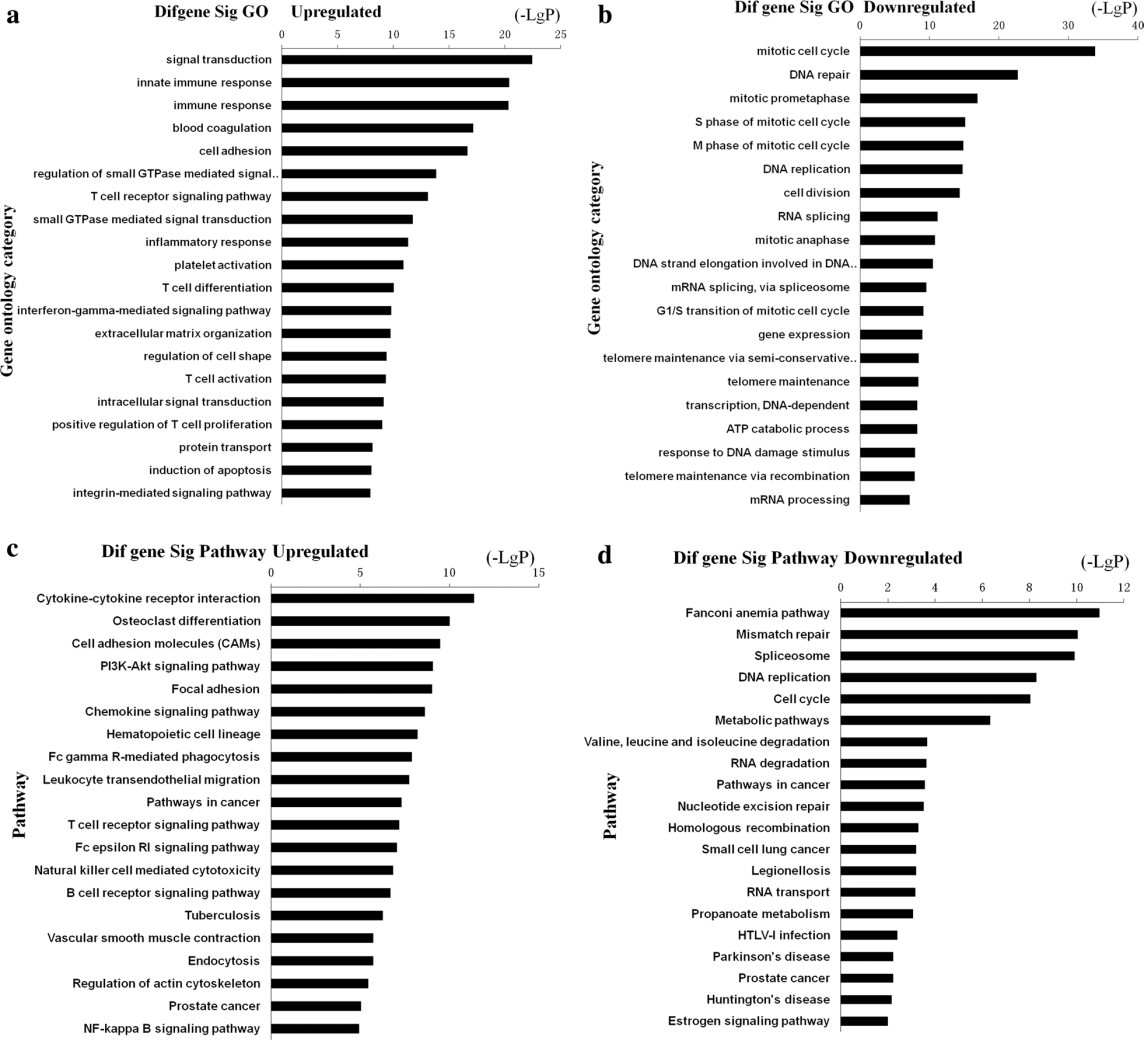
**a**, **b** Top 20 enrichment of GO terms for differentially expressed intersection genes (the bar plot shows the enrichment scores of the significant enrichment GO terms). **c**, **d** Top 20 enrichment of pathways for differentially expressed intersection genes (the bar plot shows the enrichment scores of the significant enrichment pathways)

Predicted functions of DEGs in this study were determined by intersection mRNAs. The 915 intersection mRNAs were further analyzed by GO analysis. We analyzed the enrichment of these genes. Enrichment provides a measure of the significance of the function, and as the enrichment increases, the corresponding function is more specific, which helped us to identify GO with a more definitive functional description [26]. The results showed that the up-regulated genes were significantly associated with signal transduction, innate immune response, immune response, blood coagulation, and cell adhesion, while the down-regulated genes were mainly involved in mitotic cell cycle, DNA repairment, mitotic prometaphase, S phase of mitotic cell cycle and M phase of mitotic cell cycle (Fig. 3a, b).

Pathway analysis indicated that 87 pathways corresponded to up-regulated transcripts and up-regulated genes were mainly related to cytokine–cytokine receptor interaction, osteoclast differentiation, cell adhesion molecules (CAMs), PI3K-Akt signaling pathway and focal adhesion, while down-regulated DEGs were associated with Fanconi anemia pathway, mismatch repair, spliceosome, DNA replication and cell cycle (Fig. 3c, d).

### Co-expression network

**Fig. 4:**
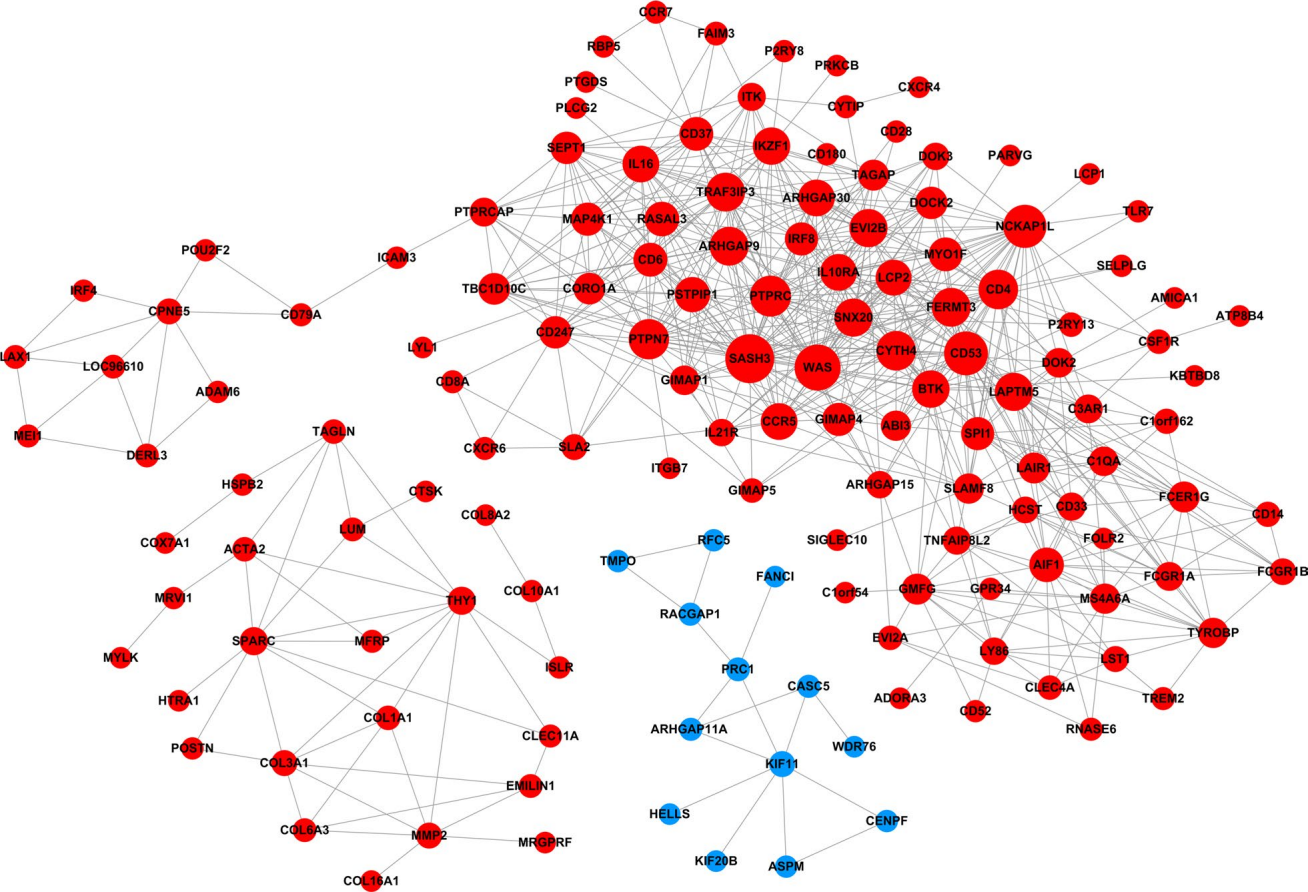
The co-expression network constructed by Cytoscape software. Proteins are represented with color nodes, and interactions are represented with edges. Red color indicated up-regulation; blue color indicated down-regulation

The intersection genes were used to construct a gene co-expression network. According to the node connectivity, genes can be further classified into hub genes. Hub genes are very important nodes and represent a small proportion of nodes with maximal information exchange with other nodes in the gene co-expression network. The entire network consisted of 145 nodes and 658 connections. Solid lines present positive correlation and dotted lines present negative correlation. The networks indicate that one gene is correlated with several genes and vice versa. A higher degree for one gene meant that the gene played a more important role in this network. Among the co-expression interactions network, there are 22 genes in the co-expression network with the degree over 20, including SASH3, WAS, CD53, NCKAP1L, PTPRC, PTPN7, CD4, CYTH4, ARHGAP9, FERMT3, TRAF3IP3, EVI2B, SNX20, LAPTM5, BTK, IKZF1, ARHGAP30, CCR5, IL10RA, IL16, LCP2 and PSTPIP1. These genes imply that they have connections with many other gene nodes (Fig. 4).

### Survival analysis

**Fig. 5:**
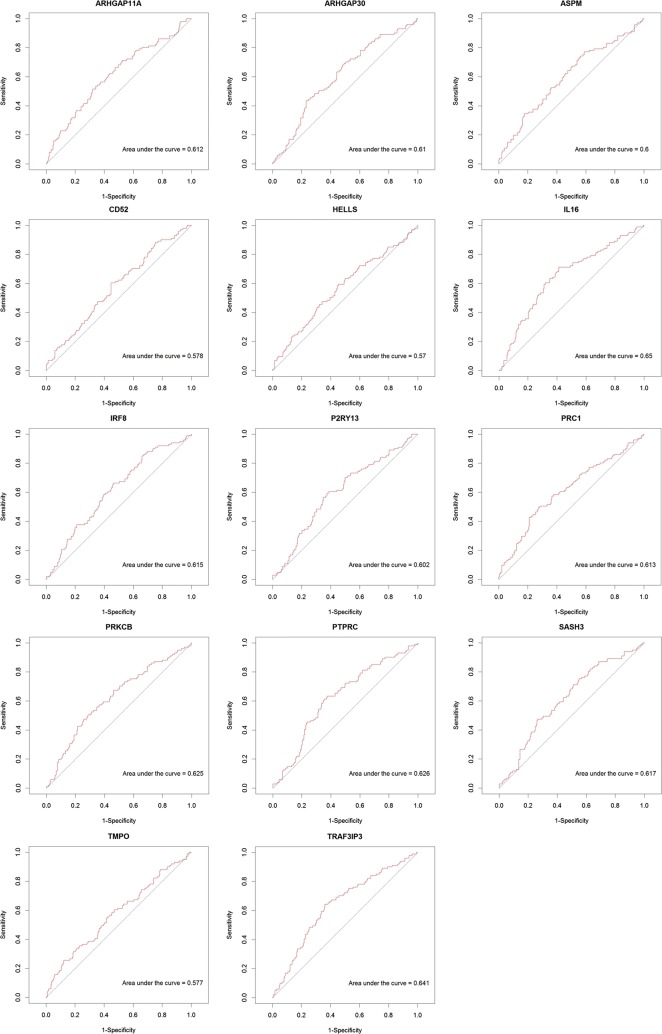
Kaplan-Meier (KM) survival curves for 14 target genes. KM survival curves show significant OS or RFS survival differences between higher-expression levels and lower-expression levels of LUAD patients

In the survival analysis, 114 genes were found to be significant in LUAD patients (log-rank test, P < 0.05). While we choose 14 target genes which were obtained by combining significant survival associated genes and genes incorporated into the gene co-expression network. In order to reveal association between the 14 target genes expression levels and LUAD prognosis, we performed Kaplan–Meier survival curves. The results show that patients with lower expression levels of five genes including ARHGAP11A, ASPM, HELLS, PRC1 and TMPO have better survival prognoses than those with higher expression levels of these five genes in LUAD. Patients with higher expression levels of the rest nine genes (ARHGAP30, CD52, IL16, IRF8, P2RY13, PRKCB, PTPRC, SASH3, TRAF3IP3) were associated with poor survival in patients with LUAD (all log-rank P < 0.05) (Fig. 5).

### ROC curve analysis

**Fig. 6:**
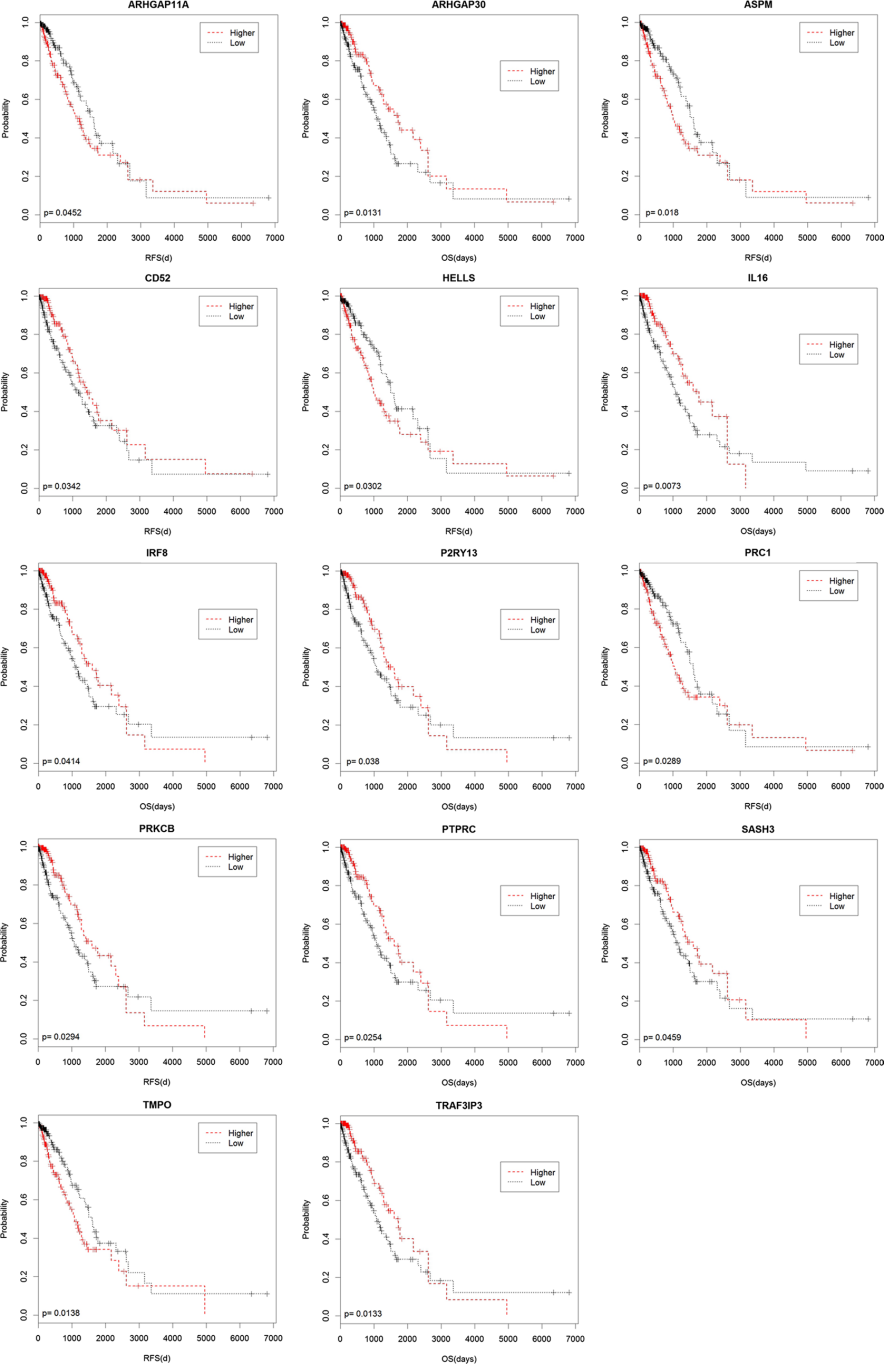
The receiver operating characteristic (ROC) curves for 14 target genes. The area under the curve (AUC) under binomial exact confidence interval was calculated to generate the ROC curve

In order to assess the discriminatory ability of the 14 target genes among LUAD metastasis and non-metastasis samples generated from TCGA database, ROC curve analyses were conducted and AUC were calculated. As Fig. 6 shown, the AUC of 11 target genes (ARHGAP11A, ARHGAP30, ASPM, IL16, IRF8, P2RY13, PRC1, PRKCB, PTPRC, SASH3, and TRAF3IP3) were more than 0.6. The AUC of CD52, HELLS and TMPO was respective 0.578, 0.57 and 0.577, less than 0.6. IL16 had the largest AUC in those 14 target genes.

### MiRNA-mRNA-lncRNA network

**Fig. 7:**
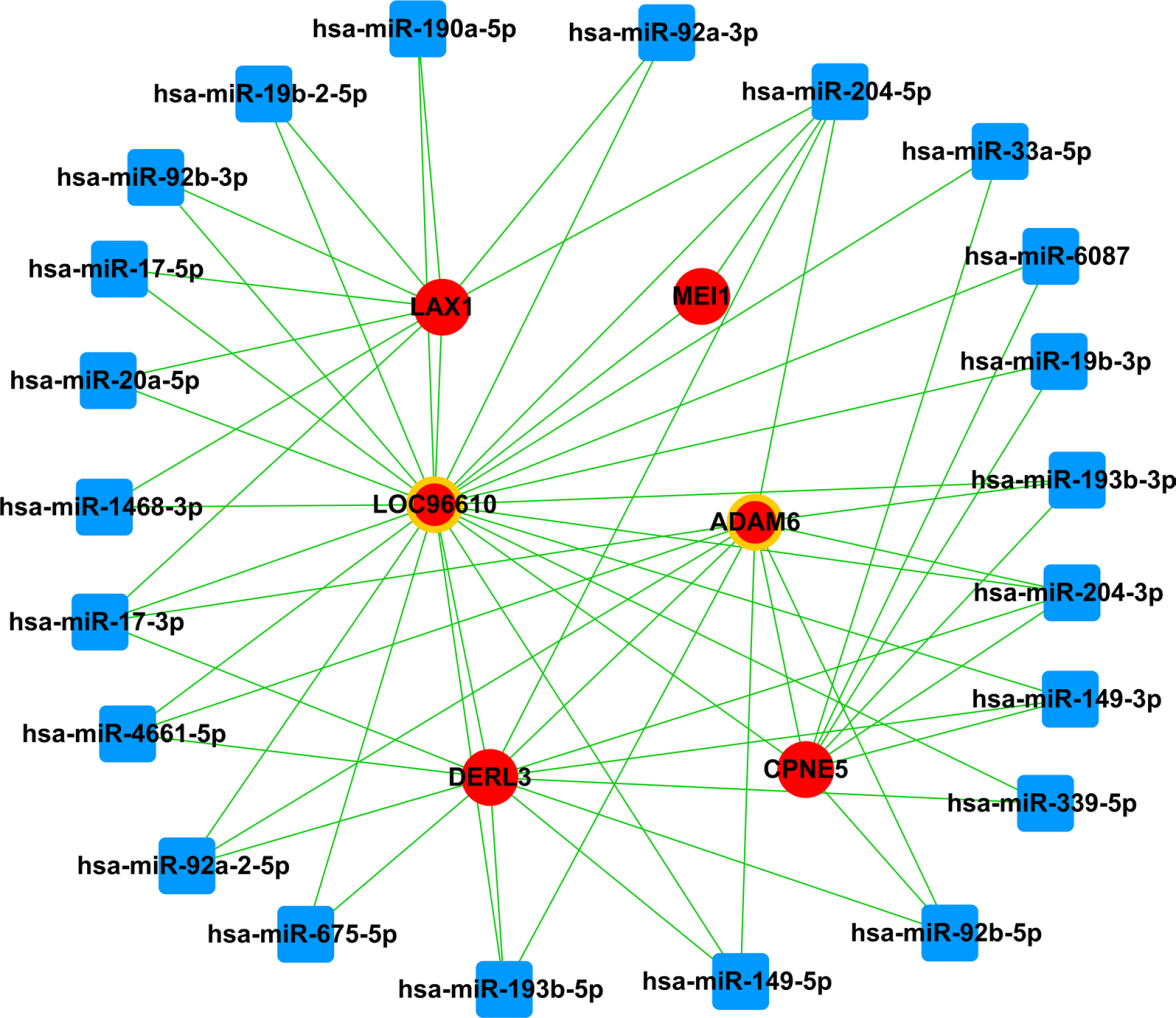
The miRNA-mRNA-lncRNA ceRNA network. Red balls represent up-regulated mRNAs, blue diamonds, down-regulated miRNAs, and red balls surrounded by yellow rings, up-regulated lncRNAs

Besides the 14 target genes which were obtained by combining significant survival associated genes and genes incorporated into the gene co-expression network, we also performed miRNA-mRNA-lncRNA to identify lncRNAs which acted as ceRNAs in LUAD pathogenesis. The miRNA-mRNA-lncRNA relationship was integrated into the ceRNA network through negative regulation. Based on the interaction network of miRNA-mRNA, miRNA-lncRNA and lncRNA-mRNA, we obtained 37 feed-forward loop networks and constructed general miRNA-lncRNA-mRNA feed-forward loop network (data not shown). In our study, the results identified that 2 lncRNAs (LOC96610 and ADAM6), 22 miRNAs and 4 mRNAs (LAX1, DERL3, MEI1 and CPNE5) were involved in the ceRNA network. The functions of LOC96610 and ADAM6 acting as ceRNAs were predicted through pathway analysis of 4 mRNAs (LAX1, DERL3, MEI1 and CPNE5) in the miRNA-lncRNA-mRNA interaction network. The results indicated that four mRNAs participated in three upregulated pathways which involved in immune response, endoplasmic reticulum unfolded protein response and male meiosis I. As a consequence, it was believed that compared with the other lncRNAs, these two lncRNAs (LOC96610 and ADAM6) might played more significant functions in the whole ceRNA network (Fig. 7).

## Discussion

Even though there was a new development of immunotherapy and targeted therapy of tumors, once the systematic metastasis of cancer cell occurred, the 5-year survival rate would decrease at less by 10% [27], and the survival of LUAD is far from satisfactory. Early diagnosis plays a critical role in the prevention and treatment of cancer, including LUAD. And researchers are continuously seeking for new biomarkers or targets for LUAD prevention, diagnose and treatment.

RNA-seq data and microarray-based expression profiling data provide a more comprehensive and accurate understanding of carcinogenesis and cancer progression at the molecular level. In our study, we analyzed the differences in the mRNAs, miRNAs, lncRNAs expression according to raw sequencing data of LUAD metastasis and non-metastasis samples from TCGA. We found that 1019 DEGs, 54 DEMs and 21 DELs are differently expressed in LUAD metastasis samples compared to non-metastasis samples. Combined with DEMs targeted mRNAs, we obtained 915 intersection mRNAs which were used to conduct GO and KEGG pathway analysis. According to the results of GO enrichment, the most significantly up-regulated genes were associated with signal transduction, while the most significantly down-regulated genes were mainly involved in mitotic cell cycle. KEGG pathway analysis showed that pathways corresponded to up-regulated transcripts were mainly related to cytokine–cytokine receptor interaction, pathways corresponded to down-regulated transcripts were mainly related to fanconi anemia pathway.

Generally, the common influence of interaction in interacting genes could not only decrease the complexity of biological network, but also benefit to explore meaningful biological information for the researchers, providing further scientific basis for therapy and study of disease [28]. In our study, the co-expression gene network based on the intersection genes provided an insight of correlation between genes. Here, we found that some genes, such as SASH3, WAS and CD53, had connections with many other gene nodes in LUAD. They might be considered as important biomarkers which account for the metastasis mechanism of LUAD. In addition, survival analysis determined that 114 genes increased or decreased expression is significantly associated with LUAD patients shorter OS or RFS, suggesting that these genes may be valuable predictive factors for LUAD patient’s survival. Finally, 14 target genes were obtained by combining significant survival associated genes and were incorporated into the gene co-expression network.

In the target genes, five genes (ARHGAP30, IL16, PTPRC, SASH3 and TRAF3IP3) were with the degree over 20, which implied the five genes not only had better important regulatory value for the network, but also had diagnostic value of LUAD metastasis. ARHGAP30, a previously uncharacterized RhoGAP domain-containing protein, as a candidate Wrch-1-binding protein. ARHGAP30 is closely related to the Cdc42-specific RhoGAP CdGAP [29], and together they form a subgroup of the RhoGAP proteins. Wang et al. [30] identified ARHGAP30 served as a key regulator for p53 acetylation, and suggested ArhGAP30 as both prognostic marker and potential therapeutic target for colorectal cancer. Besides, ARHGAP30 was found had significantly improved OS of pancreatic ductal adenocarcinoma [31]. Interleukin-16 (IL-16) is a pro-inflammatory cytokine [32] and chemo attractant for a broad variety of immune cell types with CD4 co-receptors [33]. Serum IL-16 levels have been associated with other cancers, such as multiple myeloma [34], gastric cancer [35], and colorectal cancer. Besides, IL-16 was reported to may act as a key mediator in pre metastatic niches that drives the establishment of lung metastasis and may represent a suitable therapeutic target [36]. PTPRC, also known as CD45, encodes a member of the protein tyrosine phosphatase (PTP) family, which comprises proteins commonly activated in tumors [37]. A previous report suggested that up-regulation of PTPRC resulted in high levels of inflammatory cytokines [38]. It is reported that PTPRC was predicted to interact with CXCR4, and PTPRC might also play a role in colon cancer metastasis [39]. SASH3 encodes a signaling adapter protein, containing a SLY motif in the N-terminal region, a SH3 motif and a SAM motif in the C-terminal region [40]. SAM families of receptors are known to play a role in many developmental processes including cell migration, neuronal formation and angiogenesis [40]. Schieffer et al. [41] revealed SASH3 as a hub gene was highly correlated with diverticulitis patients compared to non-diverticulosis controls. TRAF3IP3, also known as TRAF3-interacting JNK Activating Modulator (T3JAM), was originally identified as a protein that interacts specifically with Tumor necrosis factor receptor-associated factor 3 (TRAF3) to activate JNK in human kidney cells [42]. TRAF3IP3 is expressed in the immune system and participates in cell maturation, tissue development, and immune response. Nasarre et al. [43] identified new functions of TRAF3IP3 in melanoma and angiogenesis, emphasizing its physiological relevance as a potential target for cancer therapy.

Moreover, our study identified that 2 lncRNAs (LOC96610 and ADAM6), 22 miRNAs and 4 mRNAs (LAX1, DERL3, MEI1 and CPNE5) were involved in the miRNA-lncRNA-mRNA interaction network. LOC96610, located at 22q11.22, the official symbol is BMS1P20 (BMS1, ribosome biogenesis factor pseudogene 20). As a survival-related lncRNA, BMS1P20 was found significantly correlated with the pathogenesis, development and metastasis of liver hepatocellular carcinoma [44]. Furthermore, Sui et al. [45] reported that BMS1P20 positively correlated with overall survival of LUAD. ADAM6 (ADAM metallopeptidase domain 6), located at 14q32.33. ADAM is a family of membrane proteins involved in cell–cell adhesion and cell–matrix adhesion. It is characterized by a disintegrin and metalloprotease domain with an epidermal growth factor-like region and harbors both adhesion and proteolytic domains implicated in integrin function and matrix degradation [46, 47]. It is reported that an exploratory biomarker panel derived from ADAM6 conferred prognostic utility for melanoma recurrence and death [48].

## Conclusions

In summary, our findings documented that 1019 DEGs, 54 DEMs and 21 DELs were differently expressed in LUAD metastatic samples compared with non-metastatic samples. Among these altered mRNAs, many are significantly associated with LUAD patient’s survival time, and might play critical roles in LUAD metastasis. Our study highlights the important roles of mRNAs, miRNAs and lncRNAs in LUAD metastasis and may provide useful candidates as diagnostic markers and potential targets for LUAD therapy. The present study also has a few limitations, for example, the data used were obtained from TCGA, rather than directly from LUAD patients, thus a series of verification experiments must be performed to confirm our results. Overall, our findings will improve our understanding of the molecular mechanisms of LUAD and aid in finding potential targets for diagnostic and therapeutic usage.

## Additional files

**Additional file 1.** Clinical data of LUAD from TCGA cohort.

**Additional file 2.** DEGs in LUAD metastasis samples compared with non-metastasis samples.

## Abbreviations

LUAD: lung adenocarcinoma
DEGs: differentially expressed genes
DEMs: differentially expressed miRNAs
DELs: differentially expressed lncRNAs
GO: gene ontology
KEGG: Kyoto Encyclopedia of Genes and Genomes
TCGA: The Cancer Genome Atlas
FDR: false discovery rate
ROC: receiver operating characteristic
DAVID: Database for Annotation, Visualization and Integrated Discovery
AUC: area under the curve
OS: overall survival
RFS: recurrence free survival
CAMs: cell adhesion molecules
